# Effect of fear of missing out on learning burnout in medical students: a moderated mediation

**DOI:** 10.3389/fpsyt.2023.1289906

**Published:** 2023-11-17

**Authors:** Xin Ye, Yang Li, Yang Liu, Qiuyue Zheng, Zhongli Lin, Yinhua Zeng, Ziyue Lin, Tan Zhu, Xiayan Chen, Liangliang Chen, Tao Liu

**Affiliations:** ^1^School of Health, Fujian Medical University, Fuzhou, China; ^2^Mental Health Education Center, Huizhou Health Sciences Polytechnic, Huizhou, China; ^3^Department of Educational Psychology, The Chinese University of Hong Kong, Hong Kong, China; ^4^School of Psychology, Fujian Normal University, Fuzhou, China; ^5^Guidance Center for Mental Health of Students, Fujian Medical University, Fuzhou, China; ^6^Department of Developmental and Behavioral Pediatrics, Fujian Children's Hospital (Fujian Branch of Shanghai Children's Medical Center), Fuzhou, China; ^7^School of Management, Shanghai University, Shanghai, China; ^8^School of Management, Zhejiang University, Hangzhou, China

**Keywords:** fear of missing out, mobile phone addiction, sleep quality, learning burnout, mindfulness

## Abstract

**Introduction:**

Learning burnout has a significant negative impact on students’ academic performance and professional development, which has been exacerbated by the growing trend of problematic smartphone use, such as smartphone addiction, among young people. Recently, the literature on excessive social media use has revealed a critical role of fear of missing out. Objective The purpose of this study was to examine how fear of missing out affects smartphone addiction and its subsequent effect on learning burnout in college students.

**Methods:**

In Study 1, 352 medical students were recruited to complete a cross-sectional survey. In Study 2, 2,948 college students were recruited to complete a cross-sectional survey. Further in Study 3, 30 medical students were recruited into a mindfulness-based intervention program.

**Results:**

Study 1 preliminarily confirmed that fear of missing out was positively correlated with learning burnout. Study 2 then revealed a moderated mediation model showing that fear of missing out may increase smartphone addiction, which in turn affects their sleep quality and finally leads to learning burnout. This chain mediation model was moderated by the participants’ level of mindfulness. To confirm the promoting role of mindfulness, Study 3 further confirmed that mindfulness training indeed can improve smartphone addiction and reduce learning burnout in medical students.

**Discussion:**

Theoretical and practical contributions were discussed, highlighting the effects of fear of missing out on smartphone addiction and a moderating role of mindfulness training.

## Introduction

With the development of mobile internet and social media, smartphones have become an integral part of the lives of people of all ages around the world (1, 2). According to statistics, the number of smartphone subscriptions worldwide has reached 6.26 billion as of December 2021, with a penetration rate of 67% of the world’s population (3). In this context, the phenomenon of PSU is attracting increasing attention. Smartphone addiction refers to “the inability of individuals to regulate their smartphone use, which leads to negative consequences and clinical impairment in everyday life” (4). Previous studies have reported that the prevalence of smartphone addiction among college students ranges from 10% to 46% in many countries (5, 6), and this rate is rapidly increasing during the COVID-19 pandemic (7, 8). Not only can smartphone addiction lead to poor health outcomes, including physical, psychological and social problems (9–12), but it can also impair academic performance in young people. For example, college students who are addicted to their smartphones may experience lower levels of life satisfaction (2)and academic failure in college (1). Therefore, it is critical to explore the factors that induce smartphone addiction and its negative effects on academic performance among college students.

With the popularity of smartphones among the student population, problematic smartphone use (PSU), including mobile phone addiction, has become another pressing issue for Chinese medical students, with a prevalence of over 35% (5). Mobile phone addiction is characterized by excessive or compulsive use of the Internet, leading to behavioral dependence (13). The obsession and overuse of cell phones clearly affects the physical and psychological health and social functioning of adolescents (12). Students with PSU tend to have poorer self-control (14), higher perceived stress, and poorer academic performance (2), all of which may lead to student disengagement and the onset of learning burnout. For example, Rostami and colleagues reported that smartphone addiction may make students more likely to lose interest in learning and finally lead to learning burnout (15). Burnout refers to a state of exhaustion and cynicism about work (16). Accordingly, learning burnout represents the feeling of exhaustion in the learning process, which is accompanied by a cynical and detached attitude toward schoolwork, as well as a low sense of achievement (17, 18). Past studies have shown that the prevalence rate of learning burnout ranges from 45% to 71% among students worldwide (19), which is more severe among medical students (20, 21).

Due to the highly specialized requirements of the healthcare industry, medical students often face with a heavy academic workload (22, 23), require considerable time and effort to acquire the necessary professional knowledge and skills (24), and often even experience sleep deprivation (25). As a result, learning burnout tends to manifest early in medical school (26) and increases with years of medical school (27). Learning burnout may not only reduce medical students’ well-being and academic performance (28, 29), but more importantly, it may also affect their professional development and lead to higher attrition rates (19, 30–32). For example, past research has reported that learning burnout often persists into the clinical phase (26, 33), leading to a higher likelihood of medical errors (31) and ultimately affecting the quality of health care (32). Therefore, this study focused on medical students’ learning burnout to explore its relationships with smartphone addiction and the intervention.

Hamilton and colleagues have found that 64.3% of medical students with smartphone addiction had physical problems such as headaches, sleep disturbances, earaches and emotional problems such as irritability (34). In particular, a number of studies have consistently shown a positive association between smartphone addiction and poor sleep quality (9, 10, 13, 35, 36). For example, a cross-sectional study showed a positive association between smartphone addiction and bedtime procrastination among Chinese college students (37). In addition, Chung et al. found that the only significant difference in activity between low and high bedtime procrastination groups among young people during the 3 h before bedtime was smartphone use, while computer and television use were not significant (38). Consistently, Cain and Gradisar (39) suggest that smartphone backlighting has a disruptive effect on circadian rhythms, which may lead to irregular sleep schedules, and therefore smartphone addiction may lead to a reduction in sleep duration.

Sleep is a cyclical state of rest for the body and nervous system that is critical to the learning, performance, and health of college students (40, 41). Sleep not only affects cognitive processes, but is also key to recovery from stress and the elimination of fatigue (42). Previous literature has reported that irritability, chronic fatigue, difficulty concentrating, depressed mood, unsatisfactory academic progress, and academic failure are common among students with sleep disorders (43, 44). Medical students are often thought to have less free time, longer courses, longer working hours and also poorer sleep quality than most of their non-medical peers (45). Numerous studies have shown a direct correlation between sleep quality and academic burnout (46, 47). For example, Shad and colleagues found that exhaustion, a component of academic burnout, was significantly associated with poor sleep quality and daytime dysfunction due to insomnia (45). Consistently, sleep deprivation and sleepiness may increase the likelihood of poor academic performance and burnout (48, 49). Arbabisarjou et al. reported that sleep quality was a predictor of academic burnout in medical students (50), which was also found by Brubaker and Beverly during the COVID-19 pandemic (51). Therefore, smartphone addiction may affect students’ sleep quality, which in turn may lead to academic burnout.

Previous studies have made great efforts to investigate the subsequent effects of smartphone addiction, but few studies have examined its antecedent factors. Recently, a new phenomenon known as Fear of Missing Out (FoMO) has received increasing attention and has been shown to be associated with problematic behaviors in everyday life, such as excessive bitcoin investment (52) and excessive social media use (53). In particular, Li and colleagues (19–21) found that high levels of FoMO appear to contribute to smartphone addiction (54). Fear of missing out is a diffuse anxiety that arises from the fear of missing out on meaningful experiences of others and is characterized by a desire to constantly know what others are doing (55). Self-determination theory suggests that effective self-regulation and psychological well-being depend on the satisfaction of three basic psychological needs: Esteem, competence, and relatedness (56). Fear of missing out results from a lack of relatedness needs (48), and individuals with high FoMO tend to seek connection with others through specific channels or platforms to achieve basic psychological need satisfaction (57, 58). With the rise of mobile smart network devices and the shift of FoMO from traditional offline contexts to online contexts, FoMO may lead individuals to rely on smartphones as the primary means of portable online social interaction for psychological need satisfaction (59, 60), leading to the risk of misuse (61). For example, higher FoMO has been shown to be associated with greater engagement in social media use (62, 63) and greater likelihood of PSU (64–66). FoMO is strongly associated with motivation and academic performance in college students (53, 67), and FoMO is a direct predictor of academic performance, with higher levels of FoMO associated with poorer academic performance (68). For example, a recent study reported a positive correlation between FoMO and impaired cognitive performance, which may lead to poor academic performance (69) and further influence academic burnout in medical students (70, 71). In line with the research on FoMO, smartphone addiction and learning burnout, it appears that fear of missing out may exacerbate smartphone addiction among college students, which may finally lead to learning burnout.

Mindfulness can be defined as a psychological trait that refers to an individual’s tendency to remain focused in daily life (72, 73), which may provide self-help strategies for coping with stress (74) and allow individuals to disengage from automatic thoughts, habits and unhealthy behavioral patterns (72). For example, previous evidence suggests that mindfulness is negatively associated with the propensity to engage in addictive behaviors, including substance use (75, 76), perceived stress (77), anxiety (78), and craving (79). In particular, Liu et al. found a positive association between perceived stress and problematic use via self-control, which was moderated by mindfulness (80). That is, stress may lead to self-control deficits that promote problematic use when individuals have low levels of mindfulness. Consistent with this, Regan et al. reported that the effect of phobia without mobile phone on PSU decreased as mindfulness increased (81). Stratton et al. also found that for high mindfulness individuals, mindfulness significantly moderated the relationship between PSU and anxiety and stress (82).

On the other hand, previous studies have found that people with higher levels of FoMO are more worried about missing out on pleasurable experiences (65), a typical concern for the future, whereas the idea and state of focusing on the present moment promoted by mindfulness is the exact opposite. Mindfulness involves focusing attention on the present moment while adopting a curious, open, and accepting orientation toward immediate experience (83), which alleviates depression and anxiety caused by excessive focus on the past or future (84). In addition, FoMO is a need and concern for social connection, which appears to be related to insecure attachment (attachment style) (62). Increased levels of mindfulness have been shown to lead to a potential reduction in some behaviors considered characteristic of attachment insecurity, such as experiential avoidance (85). Mindfulness may reduce the likelihood that those who lack access to attachment objects will seek out some undesirable attachment alternatives (e.g., substances, gaming, and/or the Internet) (86), suggesting that high levels of mindfulness may prevent harmful use among those who would be in an emotional state of anxiety due to lack of access to a smartphone. Thus, mindfulness may moderate the relationship between FoMO and smartphone addiction and, in turn, reduce learning burnout among medical students.

**Figure 1:**
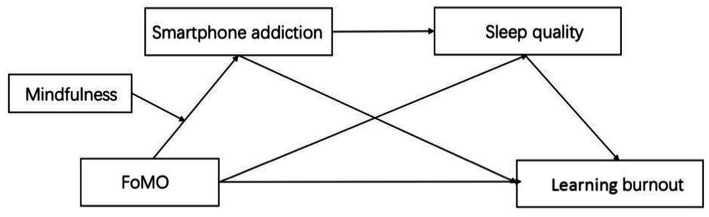
Conceptual model.

This study adopted a mixed-methods design, including two cross-sectional questionnaires and a 4-week mindfulness intervention. Study 1 first explored the effect of FoMO on learning burnout among medical students. Study 2 then examined a moderated mediation model (as shown in Figure 1), highlighting the mediating role of smartphone addiction and sleep quality and the moderating role of mindfulness. Study 3 further aimed to verify that whether or not mindfulness training could reduce smartphone addiction and learning burnout among medical students. This study has two main contributions. Theoretically, we explored the antecedent factor of smartphone addiction focusing on FoMO and revealed its impact on learning burnout, deepening the understanding of smartphone addiction among medical students. Practically, we found that mindfulness intervention could reduce the level of smartphone addiction and its negative effects, which is crucial for training qualified medical personnel for universities and society.

## Present study

To assess the proposed conceptual model, this research was conducted in three parts. First, in Study 1, we used Fear of Missing Out Scale (FoMOS) and Learning Burnout Scale (LBS) to preliminarily examine the effects of medical students’ FoMO on learning burnout. Then, based on the finding of Study 1, our sampling range and quantity were further expanded in Study 2, the Chinese version of the Smartphone Addiction Scale (SAS), Pittsburg Sleep Quality Index (PSQI) and Five Facet Mindfulness Questionnaire (FFMQ) were added to further explore the potential mechanism. Finally, according to the model structured in study 2, an intervention study was conducted to preliminarily investigate the effectiveness of a mindfulness intervention in enhancing mindfulness, improving smartphone addiction and reducing learning burnout in Study 3. All the studies were conducted in accordance with the Declaration of Helsinki, and approved by the Biomedical Research Ethical of Fujian Medical University (protocol code FJMUBRERC2021201). Informed consent was obtained from all subjects involved in the research.

### Study 1

To preliminarily confirm the relationship between FoMO and learning burnout among students majoring in medicine, a survey was administered within the medical student cohort.

#### Sample and procedures

A cross-sectional survey was conducted on an online survey platform “Questionnaire Star”,^1^ and a total of 352 medical students were recruited from Fujian Medical University in May 2021. Before starting the questionnaire, students were assured of confidentiality that the survey process would not include sensitive topics or private information, and that the results would be used only for research. The inclusion criteria for this study were participants aged between 18 and 30 years who were all college students majoring in medicine. The exclusion criteria were that (1) failed to answer both of the attention-check items (e.g., choose option A for this item) correctly, or (2) questionnaire scores greater than or less than the mean by three standard deviations. Of these, 9 (2.6%) participants were excluded, and 343 (97.4%) participants were remained in the subsequent analyses.

#### Measures

##### Fear of missing out scale (FoMOS)

The Fear of Missing Out Scale (FoMOS) is a measure designed to assess individuals’ levels of anxiety regarding missing out on social activities or information. FoMOS aims to gauge the apprehension people may experience in the era of social media, specifically fearing the possibility of missing out on experiences related to interactions with others, social events, or news events. Originally developed by Przybylski et al., FoMOS has been validated for reliability and effectiveness through multiple studies (53, 65). In 2019, Ma and Liu introduced FoMOS and provided a Chinese revision (87). In the study conducted by Ma and Liu, the fear of missing out was contextualized within “WeChat, QQ, and Spaces or Weibo,” aligning more closely with the actual situation of Chinese social networks. Specific items include, “During my holidays, I continuously and closely follow the activities of my friends through social networks such as WeChat Moments, QQ Spaces, or Weibo.” The scale consists of 10 items, utilizing a Likert 5-point scoring system (1 “Strongly Disagree” to 5 “Strongly Agree”). Confirmatory factor analysis resulted in *x*/df = 1.32, CFI = 0.94, NFI = 0.92, GFI = 0.94, TLI = 0.91, RMSEA = 0.06, SRMR = 0.03, with factor loadings ranging from 0.5 to 0.8, indicating good structural validity of the scale in the Chinese context. A higher total score indicates a higher level of fear of missing out. The Cronbach’s alpha coefficient was 0.75.

##### Learning burnout scale (LBS)

The Learning Burnout Scale (LBS) is a widely used and reliable measurement tool for assessing learning burnout among Chinese university students. It has been referenced in a substantial number of 1704 academic publications found on the China National Knowledge Infrastructure (CNKI). The LBS was administered to 672 Chinese university students by Lian et al. (88). It encompasses three aspects of learning burnout in college students: emotional exhaustion, inappropriate behavior, and low sense of achievement. The scale provides items such as “I feel exhausted after a whole day of studying,” “I rarely plan my study time,” and “I perceive the knowledge I learn as useless.” The scale has 20 items divided into three dimensions: emotional depression (8 items), inappropriate behavior (6 items), and low achievement (6 items), utilizing a Likert 5-point scoring system (1 “Strongly Disagree” to 5 “Strongly Agree”). Scores range from 20 to 100, with higher scores indicating a higher level of learning burnout. The overall Cronbach’s alpha coefficient of 0.87. The alpha coefficients for the three dimensions of emotional depression, inappropriate behavior, and low achievement were 0.81, 0.70, and 0.73, respectively, which shows that the LBS exhibits good internal consistency reliability and structural validity.

#### Statistical analyses

The data were analyzed in the following steps: first, the overall profile of learning burnout among medical students was analyzed using descriptive statistics. Second, differences in learning burnout by gender and grade-level were examined using independent samples *t*-tests or one-way ANOVAs. Finally, a linear regression analysis was used to test the relationship between FoMO and learning burnout. All statistical analyses were performed using SPSS version 26.0.

#### Results

##### Sample characteristics and preliminary analyses

Within the sample of 343 medical students analyzed, 123 (35.9%) were male and 220 (64.1%) were female. In terms of academic progression, the majority, 321 (93.3%), were classified as Freshman (1st grade). This was followed by Sophomore (2nd grade) students, representing 15 (4.4%) of the sample, and Junior (3rd grade) students accounting for 8 (2.3%). The sample characteristics and differences in learning burnout between the groups are shown in Table 1. Utilizing an independent samples *t*-test, there were no significant differences in learning burnout among medical students based on gender [*t* (341) = 1.018, *p* = 0.310]. Additionally, a one-way ANOVA revealed that the differences in learning burnout across different grade levels were also not significant [*F* (2,341) = 0.745, *p* = 0.476].

**Table 1:** Sample characteristics and the difference of learning burnout between groups.

Variables		*n* (%)	*M* (*SD*)	*t* (*F*)	*p*
Gender	Male	123 (35.9)	63.36 (6.586)	1.018	0.310
	Female	220 (64.1)	62.65 (5.874)		
Grade	1st grade	321 (93.3)	62.85 (6.165)	0.745	0.476
	2nd grade	15 (4.4)	64.73 (5.161)		
	3rd grade	8 (2.3)	62.13 (6.534)		

##### Effects of FoMO and learning burnout

To explore the relationship between FoMO and learning burnout, a linear regression analysis was conducted, using FoMO as the independent variable, and learning burnout as the dependent variable. The results in Table 2 revealed a positive correlation between fear of missing out and learning burnout (Beta = 0.266, SE = 0.065, *p* < 0.001).

**Table 2:** Regression analysis for effects of FoMO on LB.

Predictors	LB	
	*b*	*t*
Constant	55.535	30.385^***^
FoMO	0.266	4.101^***^
*R^2^*	0.047	
*F*	16.821^***^	
Adjusted *R*^2^	0.044	
Δ*R^2^*	0.047	

*N* = 343. FoMO, Fear of Missing Out; LB, Learning Burnout. ***p < 0.001.

This study provides preliminary evidence of the relationship between FoMO and learning burnout. However, the underlying mechanism of this relationship and potential avenues for addressing this issue warrant further investigation.

### Study 2

In order to gain further insights into the underlying mechanism through how FoMO influences academic burnout among college students, and building upon the findings of Study 1, an investigation into factors such as mindfulness, smartphone addiction, and sleep quality was incorporated in Study 2.

#### Sample and procedures

In this follow-up study, we used the same cross-sectional methodology as in Study 1 which was conducted on an online survey platform “Questionnaire Star” (see text footnote 1). However, in contrast to Study 1, study 2 recruited 3,085 college students in Fujian and Guangdong provinces in April 2022, which was delayed by nearly 1 year compared to Study 1 because of the COVID-19 pandemic. The inclusion criteria were still participants aged between 18 and 30 years who were all university students. The exclusion criteria were that (1) failed to answer both of the attention-check items (e.g., choose option A for this item) correctly, or (2) questionnaire scores greater than or less than the mean by three standard deviations. Of these, 137 (4.4%) participants were excluded, and 2,948 (95.6%) participants were finally remained in the subsequent analyses.

#### Measures

As an extension of Study 1 to further investigate the underlying mechanism, the same Fear of Missing Out Scale (10 items) and the Learning Burnout Scale (20 items) as used in Study 1 were adopted without modification. In addition, we included the following three additional scales:

##### Chinese version of the smartphone addiction scale (SAS)

The Smartphone Addiction Questionnaire was developed by Kwan et al. (89) and was introduced, translated and revised into Chinese by Zhao et al. (90). The scale has 33 items and 6 dimensions: interference in daily life (5 items), emotional appeasement (4 items), withdrawal (7 items), importance (4 items), cyberspace-oriented relationships (6 items), and overuse and tolerance (7 items). These items were scored on a 6-point Likert scale (1 “strongly disagree” to 6 “strongly agree”), with scores ranging from 33 to 198, with higher scores indicating more severe smartphone addiction. The Cronbach’s alpha coefficient for this scale was 0.954.

##### Pittsburg sleep quality index (PSQI)

The Pittsburgh Sleep Quality Index was developed by Buysse et al. (91) and was introduced, translated and revised into a Chinese version by Liu et al. (92). The scale consists of a total of 24 items and 7 dimensions: subjective sleep quality, time to sleep onset, sleep duration, sleep efficiency, sleep disturbance, hypnotic medication and daytime functioning. The scores for each component (ranging from 0 to 3) are accumulated to give a total PSQI score. The total score ranges from 0 to 21, with lower scores indicating better overall sleep quality. The scale had a Cronbach’s alpha coefficient of 0.823.

##### Five facet mindfulness questionnaire (FFMQ)

The Five Facet Mindfulness Questionnaire was developed by Baer et al. (93) and was introduced, translated and revised into a Chinese version by Deng et al. (94). The scale has 39 items and 5 dimensions: observing (8 items), describing (8 items), acting with awareness (8 items), not judging inner experience (8 items), and not reacting to inner experience (7 items). These items were scored on a 5-point Likert scale (1 “not at all” to 5 “completely”), with scores ranging from 39 to 195, with higher scores indicating higher levels of mindfulness. The Cronbach’s alpha coefficient for each dimension of the scale ranged from 0.732 to 0.882.

#### Statistical analyses

Data analysis was conducted in the following steps. First, to investigate potential common method variance, we conducted a principal component analysis. All self-report variables were included in this analysis. Then, independent samples *t*-tests were used to test differences on the main variables between non-medical students and medical majors, and Pearson correlation analysis was used to test for correlations between the variables. Next, the PROCESS macromodel Model 6 of SPSS with bootstrap (95) was used to examine the chain mediating role of smartphone addiction and sleep quality in the process of FoMO affecting learning burnout. Afterwards, the PROCESS macromodel Model 92 was used to examine the possible moderating effects of mindfulness levels on the chain mediating model of misplaced fear and learning burnout. Finally, the PROCESS macromodel Model 83 was used to examine the moderating role of the mindfulness levels in the effect of FoMO on smartphone addiction. To test the significance of the conditional indirect effects of Models 92 and 83, 95% bias-corrected Bootstrap confidence intervals (CIs) were constructed based on a random sample of 5,000. All statistical analyses were performed with SPSS version 26.0.

#### Results

##### Detection of common method bias

The results of the principal component analysis showed that the first principal component accounted for only 19.556% of the variance. This suggests that no single factor dominates the variance, indicating that common method variance is unlikely to be a major problem.

##### Sample characteristics and preliminary analyses

Of the 2,948 university students, 739 (25.1%) were male and 1840 (62.4%) were medical students. The sample characteristics and the differences in each of the main variables between majors (non-medical vs. medical) are shown in Table 3. Using an independent samples *t*-test, we found significant differences between medical and non-medical majors for all variables, including smartphone addiction [*t* (2946) = −9.736, *p* < 0.001], sleep quality [*t* (2946) = −2.501, *p* = 0.012], learning burnout [*t* (2946) = −4.006, *p* < 0.001] and, mindfulness [*t* (2946) = 2.247, *p* = 0.025], with the exception of FoMO [*t* (2946) = 0.093, *p* = 0.926].

**Table 3:** Differences of primary outcome variables between majors.

Variables		*M* (*SD*)	*t*	*p*
FoMO	Non-medical	24.001 (7.003)	0.093	0.926
	Medical	23.977 (6.724)		
SA	Non-medical	102.698 (27.135)	−9.736	<0.001
	Medical	112.504 (26.091)		
SQ	Non-medical	4.894 (2.786)	−2.501	0.012
	Medical	5.155 (2.726)		
LB	Non-medical	55.971 (10.154)	−4.006	<0.001
	Medical	57.513 (10.096)		
M	Non-medical	116.269 (9.252)	2.247	0.025
	Medical	115.492 (8.985)		

FoMO, Fear of Missing Out; SA, Smartphone Addiction; SQ, Sleep Quality; LB, Learning Burnout; M, Mindfulness.

##### Descriptive statistics and correlation coefficients

The means (M) and standard deviations (SD) for all variables are displayed in Table 4. The results of the Pearson correlation analysis are also shown in Table 4. As expected, there were positive correlations between FoMO and smartphone addiction (*r* = 0.401, *p* < 0.001), FoMO and learning burnout (*r* = 0.246, *p* < 0.001), smartphone addiction and learning burnout (*r* = 0.473, *p* < 0.001), sleep quality and FoMO (*r* = 0.207, *p* < 0.001), sleep quality and smartphone addiction (*r* = 0.291, *p* < 0.001), and sleep quality and learning burnout (*r* = 0.314, *p* < 0.001). There were negative correlations between mindfulness levels and all other variables (*r*s = −0.149 ~ −0.509, *p*s < 0.001).

**Table 4:** Descriptive statistics and correlation coefficients.

	*M*	*SD*	1	2	3	4	5
1 FoMO	23.99	6.829	1				
2 SA	108.82	26.906	0.401^***^	1			
3 M	115.78	9.093	−0.149^***^	−0.306^***^	1		
4 SQ	5.06	2.751	0.207^***^	0.291^***^	−0.230^***^	1	
5 LB	56.93	10.143	0.246^***^	0.473^***^	−0.509^***^	0.314^***^	1

FoMO, Fear of Missing Out; SA, Smartphone Addiction; SQ, Sleep Quality; LB, Learning Burnout; M, Mindfulness. ***p < 0.001.

##### The serial multiple mediation of smartphone addiction and sleep quality between FoMO and learning burnout

The results of the chain mediation analyses are shown in Tables 5–6. The results show that the overall effect of FoMO on learning burnout was significant, while the direct effects were not statistically significant. Based on bootstrap confidence intervals, three additional indirect effects were significant: FoMO → smartphone addiction → learning burnout, FoMO → sleep quality → learning burnout, and FoMO → smartphone addiction → sleep quality → learning burnout. The total indirect effect was 0.317 and the three indirect effects were 0.253, 0.035 and 0.028, respectively. The total indirect effect and the three indirect effects as a percentage of the total effect were 87.569%, 69.890%, 9.669%, and 7.735%, respectively.

**Table 5:** Testing the serial multiple mediation of SA and SQ between FoMO and LB.

Predictors	Model 1 (SA)		Model 2 (SQ)		Model 2 (LB)	
	*b*	*t*	*b*	*t*	*b*	*t*
FoMO	1.628^***^	25.129	0.049^***^	6.296	0.045	1.723
SA			0.024^***^	11.733	0.156^***^	22.700
SQ					0.722^***^	11.743
LB						
						
*R^2^*	0.212		0.104		0.268	
*F*	198.202^***^		68.492^***^		178.964^***^	

*N* = 2,948. Results are adjusted for gender, grade and major. Each column is a regression on model that predicts the criterion at the top of the column. FoMO, Fear of Missing Out; SA, Smartphone Addiction; SQ, Sleep Quality; LB, Learning Burnout. ***p < 0.001.

**Table 6:** Total, direct and indirect effects of FoMO on LB.

	Effect	SE	LLCI	ULCI
Total effect	0.362	0.027	0.310	0.414
Direct effect	0.045	0.026	−0.006	0.096
Indirect effects				
Total	0.317	0.019	0.281	0.355
Ind1: FoMO → SA → LB	0.253	0.017	0.222	0.288
Ind2: FoMO → SQ → LB	0.035	0.007	0.023	0.049
Ind3: FoMO → SA → SQ → LB	0.028	0.004	0.021	0.035
Ind1 minus Ind2	0.218	0.019	0.182	0.255
Ind1 minus Ind3	0.226	0.017	0.193	0.259
Ind2 minus Ind3	0.008	0.007	−0.007	0.022

*N* = 2,948. Results are adjusted for gender, grade and major. FoMO, Fear of Missing Out; SA, Smartphone Addiction; SQ, Sleep Quality; LB, Learning Burnout; SE, Standard Error; LLCI, Low level confidence interval; ULCI, Upper level confidence interval.

**Table 7:** Testing the moderation of mindfulness on FoMO → SA path of the serial multiple mediation of SA and SQ between FoMO and LB.

Predictors	Model 1 (SA)		Model 2 (SQ)		Model 3 (LB)	
	*b*	*t*	*b*	*t*	*b*	*t*
FoMO	1.491^***^	23.804	0.049^***^	6.296	0.045	1.723
SA			0.024^***^	11.733	0.156^***^	22.700
SQ					0.722^***^	11.743
LB						
Mindfulness	−0.765^***^	−16.230				
FoMO*Mindfulness	0.022^**^	3.277				
SA*Mindfulness						
SQ*Mindfulness						
						
*R^2^*	0.279		0.104		0.268	
*F*	189.544^***^		68.492^***^		178.964^***^	

*N* = 2,948. Results are adjusted for gender, grade and major. Each column is a regression on model that predicts the criterion at the top of the column. FoMO, Fear of Missing Out; SA, Smartphone Addiction; SQ, Sleep Quality; LB, Learning Burnout. **p < 0.01, ***p < 0.001.

**Table 8:** Direct and indirect effects of FoMO on LB.

	Mindfulness (*M* ± *SD*)	Effect	SE	LLCI	ULCI
Direct effect					
	–	0.045	0.026	−0.006	0.096
Conditional and unconditional indirect effects					
FoMO → SA → LB					
	−9.093	0.201	0.017	0.168	0.234
	0.000	0.232	0.016	0.201	0.264
	9.093	0.263	0.020	0.224	0.303
FoMO → SQ → LB					
	–	0.035	0.007	0.023	0.049
FoMO → SA → SQ → LB					
	−9.093	0.022	0.003	0.016	0.028
	0.000	0.025	0.003	0.019	0.032
	9.093	0.029	0.004	0.021	0.037

*N* = 2,948. Results are adjusted for gender, grade and major. FoMO, Fear of Missing Out; SA, Smartphone Addiction; SQ, Sleep Quality; LB, Learning Burnout; SE, Standard Error; LLCI, Low level confidence interval; ULCI, Upper level confidence interval.

##### The moderation of mindfulness on the serial multiple mediation of smartphone addiction and sleep quality between FoMO and learning burnout

The Model 92 of the PROCESS macro was first used to examine whether possible indirect effects of FoMO on learning burnout were moderated by mindfulness in the chain mediation model, the statistical results showed that only the interaction between FoMO and mindfulness had a significant positive predictive effect on smartphone addiction among college students, and interactions on the other paths were not significant. According to the above results, the moderated mediation model was revised, and the Model 83 of the PROCESS macro was further run to test the moderation effect of mindfulness, the statistical results of which are shown in Tables 7, 8. Taken together, these results indicated that the mediation effects on two pathways were significantly moderated by mindfulness: fear of missing out → smartphone addiction → learning burnout, and fear of missing out → smartphone addiction → sleep quality → learning burnout.

This study confirmed the mediating role of smartphone addiction and sleep quality in the chain relationship between FoMO and learning burnout. Moreover, mindfulness was found to moderate the association between FoMO and smartphone addiction, suggesting that individuals with higher levels of mindfulness may be less likely to experience smartphone addiction and learning burnout when experiencing FoMO. Thus, it appears that further exploration is warranted to examine whether alleviating these issues can be achieved by regulating individuals’ levels of mindfulness.

### Study 3

Building upon the foundations laid in Study 2, we identified mindfulness as a potential moderating variable among FoMO and learning burnout. To examine whether mindfulness can potentially alleviate issues related to smartphone addiction and learning burnout, we designed Study 3 including a 4-week mindfulness intervention to explore a viable solution for addressing learning burnout among medical students.

#### Sample and procedures

In Study 3, we recruited 30 medical students from Fujian Medical University for a 4-week mindfulness intervention between May 2022 and June 2022. Data were collected twice, before and after the intervention. The inclusion criteria for this study involved selecting the top 40 participants with the highest academic burnout scores from the 352 participants in Study 1 with high levels of willingness to participate in the mindfulness intervention course in Study 3. Exclusion criteria included: (1) participants with severe mental disorders or suicidal tendencies, (2) participants with serious physical illnesses, or (3) participants who drop out during the 4-week intervention practice. Of these, 10 (25.5%) participants were excluded in total, and 30 (75.5%) participants’ data were included in the final analysis.

#### Mindfulness training program

Participants are required to attend a group mindfulness training course of 1 h per week for 4 weeks. The course includes many classic mindfulness training practices (see Table 9). In addition, participants are required to complete at least 10 min of podcast-guided mindfulness practice each day as homework. The podcast material is taken from the book “The Mindful Way Workbook: An 8-Week Program to Free Yourself from Depression and Emotional Distress” (96). After 4 weeks, all participants were required to complete a series of questionnaires assessing their FoMO, smartphone addiction and learning burnout. The mindfulness training program was led by an experienced trainer (one of the authors) who has completed the 4-stage of MBCT (Mindfulness-based Cognitive Therapy) training offered by the Oxford Centre of Mindfulness.

**Table 9:** Activities of the mindfulness training program.

Week	Activities			
1	Mindful breathing 10 min	Mindful of all senses 25 min	Body scan 25 min	
2	Body scan 25 min	Mindful walking 15 min	Mindful stretching 10 min	Mindful sitting 10 min
3	Mindful stretching 20 min	3-min mindful breathing 5 min	Body scan 25 min	Mindful breathing 10 min
4	Mindful sitting 10 min	Body scan 25 min	Mindful walking 20 min	3 min mindful breathing 5 min

#### Measures

Levels of mindfulness, FoMO, smartphone addiction and learning burnout were assessed using the FFMQ, FoMOS, SAS and LBS from Study 2, respectively.

#### Statistical analyses

Paired samples *t*-tests were used to compare scores on levels of mindfulness, FoMO, smartphone addiction and learning burnout before and after the mindfulness intervention. All statistical analyses were conducted using SPSS version 26.0.

#### Results

As shown in Table 10, mindfulness scores increased significantly [*t* (29) = −2.569, *p* < 0.05, Cohen’s *d* = 15.705], confirming the validity of the mindfulness training. Then, scores for smartphone addiction [*t* (29) = 2.895, *p* < 0.01, Cohen’s *d* = 25.981] and learning burnout significantly decreased after the mindfulness training [*t* (29) = 3.194, *p* < 0.01, Cohen’s *d* = 6.173], whereas scores for FoMO did not significantly decrease [*t* (29) = 1.320, *p* > 0.05], suggesting that mindfulness training indeed could reduce levels of smartphone addition and improve learning burnout.

**Table 10:** Comparisons of the mindfulness, FoMO, SA and LB before and after mindfulness intervention.

	Before intervention		After intervention		*t*	*p*
	*M*	*SD*	*M*	*SD*		
Mindfulness	111.533	8.415	118.900	11.769	−2.569	0.016
FoMO	28.733	3.600	27.200	6.692	1.320	0.197
SA	132.500	22.563	118.767	19.948	2.895	0.007
LB	66.200	3.854	62.600	4.987	3.194	0.003

*N* = 30. FoMO, Fear of Missing Out; SA, Smartphone Addiction; LB, Learning Burnout.

## Discussion

The purpose of this research was to explore the potential mechanisms by which FoMO affects college students’ learning burnout through smartphone addiction and sleep quality and to further evaluate the role of mindfulness in improving college students’ smartphone addiction and learning burnout. Through two cross-sectional studies and one intervention study, we demonstrated that FoMO has a significant impact on medical students’ learning burnout, the process is mediated through the chain of smartphone addiction and sleep quality, which may be moderated by individuals’ mindfulness. Mindfulness training may be a potential intervention to reduce smartphone addiction and improve learning burnout in medical students. This study has both theoretical and practical implications.

Previous studies have focused on the relationship between FoMO and academic performance in college students. For example, Suad and Hamed investigated the level of FoMO among 2,084 undergraduate students at Kuwait University and found that the level of FoMO among university students was strongly associated with distraction and learning disengagement (97). Similarly, several studies have also found that FoMO is associated with the surface learning style, and the higher the level of FoMO, the more inclined one is to choose the surface learning style over the deep learning style (98, 99). The surface learning style is a less effective learning strategy (100), students who use a surface learning style tend to put the least effort into their learning, which may result in poor academic performance (101). Regarding the underlying mechanism, Potsaid and Venkataraman found that college students’ FoMO was associated with information overload from social media, which in turn led to poor self-regulation and ultimately lower academic performance (102). In addition, some studies have also found an association between FoMO and academic motivation and academic performance among college students (53, 67, 68). However, the direct relationship between FoMO and academic burnout has been less explored. In this research, we focus on medical students and provide preliminary evidence that FoMO may be an important factor influencing learning burnout among medical students.

Studies in the literature have shown that FoMO is strongly associated with smartphone addiction or PSU or frequent use of social media platforms (65, 103–105). Wolniewicz et al. further found that FoMO was the most important predictor of PSU variables (104). Consistent with previous findings, our results also show a positive correlation between FoMO and smartphone addiction. Regarding the subsequent effects of smartphone addiction, several studies have revealed the negative impact of smartphone addiction on academic performance (1, 106–110) and other social functions such as interpersonal relationships (111–113). However, the underlying mechanism of FoMO on learning burnout is still less examined. Our findings provide preliminary evidence that the relationship between FoMO and learning burnout is mediated by smartphone addiction and sleep quality. This research contributes to the literature on FoMO and learning burnout and deepens our understanding of learning burnout among medical students.

In recent years, there has been growing evidence for the potential role of mindfulness in promoting well-being and reducing anxiety, depression and academic stress in populations such as graduate and undergraduate students (114–116). Previous research has shown that trait mindfulness is an important resource for young people to cope with learning burnout (117, 118). In addition, mindfulness may help maintain high levels of attentional control, emotional control, and executive control (119, 120), which may further reduce the likelihood of excessive smartphone use (121). Practicing mindfulness can prevent PSU by reducing the intensity of risk factors (81). However, research on the relationship between mindfulness and FoMO is limited. Our results revealed a moderated role of mindfulness in the process of FoMO affecting learning burnout via smartphone addiction.

Mindfulness training has emerged as a new way to teach people to cope with stress more effectively (122–124), and can help people distance themselves from psychological urges, which in turn may further reduce addictive impulses and regulate the negative emotional states associated with addiction (125). Mindfulness-based Intervention (MBI) has been used in behavioral addiction research in recent years, but there are few empirical studies using MBI on the prevalence of smartphone addiction among Chinese medical students (126). Focusing on medical students, this research provides preliminary evidence that mindfulness training may help ameliorate smartphone addiction and learning burnout among medical students. This research contributes to the literature on mindfulness and smartphone addiction and learning burnout. In addition, our preliminary findings suggest that mindfulness training could be designed as a practical course to help medical students reduce their smartphone addiction, improve learning burnout, and ultimately enhance the quality of health care services in the future.

Although we have provided preliminary evidence of the role of mindfulness in FoMO and learning burnout through a large-scale survey and an intervention experiment, the current study still has several limitations. First, Study 2 was a cross-sectional study, and future longitudinal studies should be considered to explore the developmental patterns of the variables. Second, the intervention program in Study 3 lacked a control group and was only pre-and post-tested, which cannot rule out a placebo effect, limiting the validity of our findings. Third, we were also unable to answer the question of how long the beneficial effects of mindfulness training on smartphone addiction and learning burnout would last. Future studies need to replicate the mindfulness training program using a repeated measures design. Fourth, the 4-week program did not change participants’ levels of FoMO, and future studies should examine the effects of different doses and durations of FoMO. In the future, we will conduct more in-depth research focusing specifically on the role of mindfulness in these domains. Finally, it is important to note that while PROCESS macro analyses provide valuable insights into mediation and moderation effects, SEM offers the advantage of explicitly modeling latent variables and measurement error. In our study, we chose to use the PROCESS macro because of its streamlined approach to quickly validating moderated mediation effects, the direct interpretability of results, and the advantages of bootstrapping in estimating confidence intervals. However, we also recognized that SEM may provide a more comprehensive control for measurement error and latent constructs. Future research, especially where measurement error may have a significant impact on results, should consider the advantages of SEM.

## Conclusion

This research focuses on learning burnout and its contributing factors among medical students and provides preliminary evidence that individuals with high levels of FoMO may have more severe smartphone addiction problems and poor sleep quality and thus have more severe learning burnout conditions. Mindfulness training may be a potentially effective coping strategy for medical students to reduce smartphone addiction and improve learning burnout.

## Data availability statement

The raw data supporting the conclusions of this article will be made available by the authors, without undue reservation.

## Ethics statement

The studies involving humans were approved by the Biomedical Research Ethical Committee of Fujian Medical University (Protocol code FJMUBRERC2021201). The studies were conducted in accordance with the local legislation and institutional requirements. The participants provided their written informed consent to participate in this study.

## Author contributions

XY: Conceptualization, Data curation, Formal analysis, Funding acquisition, Investigation, Methodology, Resources, Writing – original draft. YLi: Investigation, Methodology, Resources, Writing – original draft, Formal analysis. YLiu: Methodology, Writing – original draft. QZ: Writing – original draft. ZhL: Writing – original draft. YZ: Writing – original draft. ZiL: Writing – original draft. TZ: Writing – original draft. XC: Writing – original draft. LC: Formal analysis, Resources, Writing – original draft. TL: Conceptualization, Formal analysis, Methodology, Resources, Writing – review & editing, Project administration.

## Footnotes

^1^ https://www.wjx.cn/
